# Age of machine learning: new trends in autism spectrum disorder prediction

**DOI:** 10.3389/fmicb.2025.1492484

**Published:** 2025-07-11

**Authors:** Weihong Xu, Haibei Li, Junwen Li, Min Jin

**Affiliations:** Tianjin Key Laboratory of Risk Assessment and Control for Environment & Food Safety, State Key Laboratory of Pathogen and Biosecurity, Academy of Military Medical Sciences, Tianjin, China

**Keywords:** autism spectrum disorder, machine learning, risk prediction model, intestinal microbiome, biomarkers

## Abstract

In recent years, there has been an increase in the incidence of autism spectrum disorder (ASD), its pathogenesis remains unknown, and there are no effective treatments available. Early identification of individuals at risk enables early targeted intervention, which improves outcomes. Through the integration of artificial intelligence and the medical field, researchers can establish a machine learning (ML) risk prediction model to estimate the risk of ASD. Currently, a variety of risk models have been developed using multiple factors, such as genetic background, gaze behavior, adverse conditions during pregnancy and childbirth, magnetic resonance imaging of the brain, and intestinal microbial composition, to predict ASD. These ML prediction models have shown some reliability in predicting ASD risk. In the future, ML prediction models for ASD will present significant challenges and opportunities, potentially helping identify drug targets for developing novel therapies to alleviate ASD symptoms and enable precision medicine.

## 1 Introduction

Autism spectrum disorder (ASD) is a common form of pervasive developmental disorder (PDD) characterized by neurodevelopmental abnormalities, resulting in communication difficulties, stereotyped actions, repetitive behaviors, and apathy (Matson et al., 2009, Santocchi et al., 2016). The incidence of ASD is high. According to the United States Centers for Disease Control (CDC) report in 2021, about 1 in 44 (2.3%) 8-year-old children were diagnosed with ASD by the Autism and Developmental Disabilities Monitoring (ADDM) Network in 2018, with a prevalence rate 3.39 times higher than that in 2000 (Maenner et al., 2021). In 2022, the Development of the Autism Education and Rehabilitation Industry estimated that the number of ASD patients in China may exceed 10 million, with over 2 million being children aged 0–14 years and nearly 200,000 new cases per year (AERI, 2022). Zeidan et al. calculated a global prevalence rate of 1:100 for autism from 2012 to 2021 (Zeidan et al., 2022). In Australia, 0.74% of children under the age of 7 are diagnosed with ASD (Bent et al., 2015). Therefore, ASD has emerged as a global public health concern.

**FIGURE 1:**
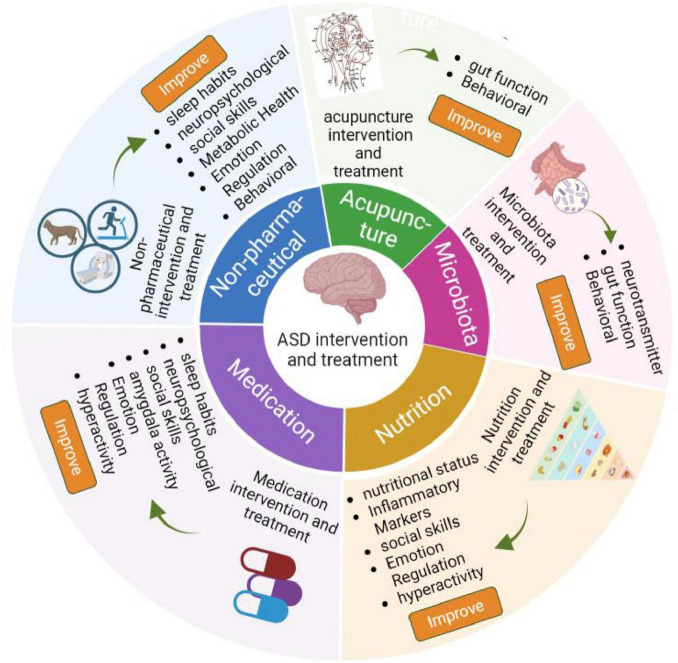
The intervention and treatment of ASD.

Nowadays, it is believed that ASD is a complex process driven by the accumulation of genetic, demographic, environmental, and adverse factors during pregnancy (Qin et al., 2024). However, the detailed pathogenesis of ASD is still poorly understood. Currently, ASD diagnosis is less than ideal, as it depends on interviews and questionnaires (Guthrie et al., 2012, Liu et al., 2019, Doi et al., 2022). Not only does it require a high level of diagnostic experience from physicians, but these methods also fail to detect child patients under 3 years old, who may have the highest detection rate due to their lack of typical autism characteristics. Failure to identify these children can disturb the development of their nervous system, resulting in lower efficacy in ASD management. Importantly, the intervention and treatment effects of ASD are also limited, which include non-pharmaceutical interventions, such as music therapy, exercise, cognitive behavioral therapy (CBT), equine-assisted activities, animal assisted therapy (Tse, 2020, Lopata et al., 2020, Rabeyron et al., 2020), targeting microbiota (Sanctuary et al., 2019, Kang et al., 2020, Wang et al., 2020), acupuncture (Chan et al., 2009, Wang et al., 2022), drug treatments (Golubchik et al., 2011, Harfterkamp et al., 2012, Scahill et al., 2015, Hendren et al., 2016, Ichikawa et al., 2016, Levine et al., 2016, Maras et al., 2018, Parker et al., 2019, Gabis et al., 2019, Ballester et al., 2019, Wichers et al., 2019, Mahdavinasab et al., 2019, Momtazmanesh et al., 2020, Soorya et al., 2021, McDougle et al., 2022, Le et al., 2022, Hacohen et al., 2022), nutrition supplements, such as tetrahydrobiopterin, resveratrol, vitamin D, omega-3, fatty acid, d-Cycloserine, folinic acid (Klaiman et al., 2013, Frye et al., 2016, Wink et al., 2017, Adams et al., 2018, Hendouei et al., 2019, Mazahery et al., 2019, Javadfar et al., 2020, Keim et al., 2022), as shown in Figure 1.

In recent years, machine learning (ML) has emerges as a key tool for early detection, diagnosis, and intervention of disease by analyzing large amounts of sample data to identify risk factors for disease. Duggan, M. R. (Duggan and Walker, 2024) utilized ML on plasma proteome data to predict 11 organ-specific aging diseases, including heart failure, cognitive decline, and Alzheimer’s Disease (AD). In addition, ML has been used to predict cardiovascular disease and breast cancer based on the human microbiome (Liu et al., 2024). In this review, we summarize the risk factors of ASD and the growing role of ML in predicting ASD, especially through the analysis of intestinal microbiome. Establishing an ML risk prediction model for ASD using gut microbiota and applying it to clinical practice may facilitate for the prediction and treatment of ASD in the future.

## 2 Risk factors of ASD

**FIGURE 2:**
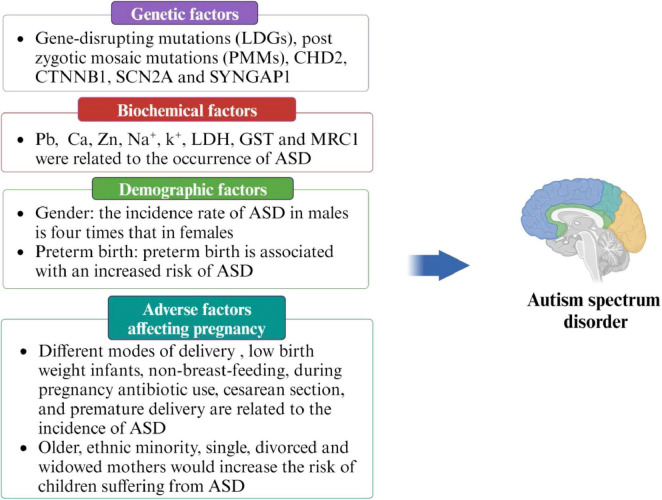
Risk factors of ASD.

ASD may be a multifactorial disease, and a single factor alone is not sufficient to fully explain its onset. Therefore, it is crucial to identify and understand the various risk factors in order to prevent the occurrence of ASD. Previous studies have identified several high-risk factors related to ASD, including genetic factors (Zhang and Shen, 2016, Krupp et al., 2017), demographic factors (Perales-Marín et al., 2021, Crump et al., 2021, Faruk et al., 2023), adverse factors affecting pregnancy (Limperopoulos et al., 2008, Darcy-Mahoney et al., 2016, Al-Zalabani et al., 2019, Hamad et al., 2019, Axelsson et al., 2019, Madany et al., 2022a,b, Sousamli et al., 2024), and biochemical factors (Wu et al., 2018, Kim et al., 2019, Hassan et al., 2022, Zhang et al., 2022). Detailed information is provided in Figure 2.

### 2.1 Demographic factors

Preterm birth and gender have been associated with an increased risk of ASD development in infants (Crump et al., 2021, Haghighat et al., 2022, Tartaglione et al., 2022). A national cohort study performed on 4,061,795 infants born in Sweden from 1973 to 2013 found that preterm birth is associated with a higher risk of ASD (Beggiato et al., 2017). This could be attributed to the neurological development of the fetus. Alfred *et al.* (Supekar et al., 2022) conducted a population-based case-control study involving 128 children diagnosed with ASD and 311 control subjects in Spain. The study utilized questionnaires to collect information and found a higher incidence of ASD among children born via cesarean section and male gender. The incidence rate of ASD in males is four times that in females (Wu et al., 2022). This disparity may be due to the underrecognition of ASD in girls by diagnostic tools and sex-related genetic factors (Wilfert et al., 2021). The brains functionally organized differently of females and males with ASD, such as motor, language and visuospatial attentional systems (Supekar et al., 2022), which shows that the different incidence rate of ASD between male and female may be related to brain function.

### 2.2 Pregnancy, delivery, and postpartum factors

Exposure to adverse risk factor during pregnancy can significantly impact the normal development of the fetus, leading to impaired neurodevelopment in children. Several factors, such as different modes of delivery, low birth weight, lack of breastfeeding, antibiotic use during pregnancy (Hamad et al., 2019, Madany et al., 2022a,b), cesarean section (Al-Zalabani et al., 2019, Axelsson et al., 2019), and premature delivery (Limperopoulos et al., 2008) has been linked to an higher incidence of ASD. A retrospective study analyzed 664 records of children treated at one of the largest ASD treatment centers in the United States from March 1, 2009, to December 10, 2010 found that children born to older mothers, as well as those from ethnic minority, or raised by single, divorced, or widowed mothers, had an elevated risk of developing ASD (Darcy-Mahoney et al., 2016). In addition, multicenter studies have shown that maternal exposure to the triclosan during pregnancy can cause ASD in offspring, significant male (Wu et al., 2022). Hamad et al. analyzed data from Manitoba’s population, including 214,834 children born in Manitoba, Canada, from April 1, 1998, to March 31, 2016 and found that children exposed to antibiotics during the second or third trimester of pregnancy had an elevated risk of developing ASD (Hamad et al., 2019).

### 2.3 Genetic factors

Genetic mutations related to ASD result in neurodevelopmental impairments (Wilfert et al., 2021). A recent study identified gene expression signatures in various neuronal cell types associated with genes containing likely gene-disrupting mutations (LGDs) in ASD patients and screened 117 risk genes (Zhang and Shen, 2016). The analysis of relevant gene subsets and genetic variations associated with non-syndromic ASD was conducted using databases such as the National library of medicine (ClinVar), autism research community focusing on genes implicated in autism susceptibility (SFARI Gene), and Autism informatics portal (AutDB). Through this analysis, a subset of twenty overlapping genes potentially specific to non-syndromic ASD was identified. These genes were found to be enriched in biological processes related to neuronal development and differentiation, synaptic function, and social behavior (An et al., 2018). Krupp e*t al.* evaluated the potential role of post-zygotic mosaic mutations (PMMs) in ASD risk and identified the risk genes CHD2, CTNNB1, SCN2A, and SYNGAP1, as well as the candidate risk genes ACTL6B, BAZ2B, COL5A3, SSRP1, and UNC79, which may be involved in chromatin remodeling or neural development (Krupp et al., 2017). These findings highlight neurogenesis, chromatin modification, and synaptic functions as key potential mediators of genetic vulnerability. Furthermore, Whole-genome sequencing (WGS) of de novo and rare inherited single-nucleotide variants (SNVs), along with structural variations in genes previously linked to ASD and other neurodevelopmental disorders, revealed important insights. In one case, two ASD-affected siblings harbored distinct ASD-related mutations, rather than sharing a common risk variant. Interestingly, these siblings exhibited greater clinical variability compared to cases where siblings shared a common risk variant (Yuen et al., 2015). This suggests that variations in ASD-associated genetic factors may contribute to clinical heterogeneity, thereby complicating ASD identification.

### 2.4 Biochemical factors

A study tested the blood of 1,537 children in Xinjiang from September 2018 to September 2019 and found that Pb, Ca, and Zn were linked to the occurrence of ASD (Zhang et al., 2022). A meta-analysis encompassed 29 case-control studies involving a total of 2,504 children with ASD and 2,419 healthy controls. The analysis revealed that copper levels in hair were significantly lower in children with ASD compared to healthy controls. However, no significant difference was observed in blood copper levels between the ASD group and the control group (Liu et al., 2023). This indicates that copper has adverse effects on the accumulation sites in the body. Detection in the hair indicates a higher accumulation in the head, which can cause significant damage to the brain. In addition, vitamin D status in neonates was significantly associated with ASD and intellectual disabilities (Wu et al., 2018). In another study, Hassan et al. analyzed blood samples from 40 ASD patients and 40 healthy controls. Their findings revealed that sodium (Na^+^), potassium (K^+^), Lactate Dehydrogenase (LDH), Glutathione-S-Transferases (GST), and Mannose Receptor C Type 1 (MRC1) were all associated with ASD onset (Hassan et al., 2022). Blood contains various ions that play a role in the regulation of nervous system activities and exchanging nutrition substances with cells through a variety of ion channels.

Research has evaluated perinatal biomarkers associated with the subsequent development of ASD. Utilizing banked cord blood plasma samples and clinical data from the Iowa Maternal Fetal Tissue Bank, the study found elevated levels of homocysteine, myristic acid, and pentadecanoic acid in ASD samples. Conversely, levels of L-isoleucine, L-threonine, O-phosphoethanolamine, and 2-hydroxybutyric acid were reduced (Brandon et al., 2022). In cytokine levels, comparing those later diagnosed with ASD (*n* = 38) to typically developing (TD) (*n* = 103) infants at 3 years of age. The results showed elevated levels of granulocyte colony-stimulating factor (G-CSF) and reduced levels of interleukin-1α (IL-1α), IL-1β, and IL-4 in the ASD group (Moreno et al., 2024). The Barwon Infant Study, which involved n = 1,074 mother-child pairs, identified a correlation between elevated cord blood acylcarnitine levels and increased Attention Deficit Hyperactivity Disorder (ADHD)/ASD symptoms at age of two. This relationship appeared to be partially mediated by socioeconomic factors such as low income, low Apgar scores, and maternal inflammation (Vacy et al., 2024). A total of 567 children (92 with ASD and 475 neurotypical) from the Boston Birth Cohort study were enrolled at birth and prospectively monitored at the Boston Medical Center. Elevated levels of cord blood unmetabolized folic acid were linked to an increased risk of ASD among Black children (Raghavan et al., 2020), underscoring the need to consider ethnic-specific factors for prenatal folic acid supplementation.

ASD is characterized by a complex interplay of risk factors, including genetic predisposition, biochemical imbalances, prenatal complications, and gut microbiota dysbiosis. While progress has been made in understanding these contributing elements, substantial gaps remain. Future research should integrate multi-omics data, such as genomic, radiomic, and microbiome, with Artificial Intelligence (AI) analytics. This system-level approach could decode ASD heterogeneity through computational modeling, paving the way toward personalized diagnostics and therapies.

## 3 Screening with ASD ML prediction model

**FIGURE 3:**
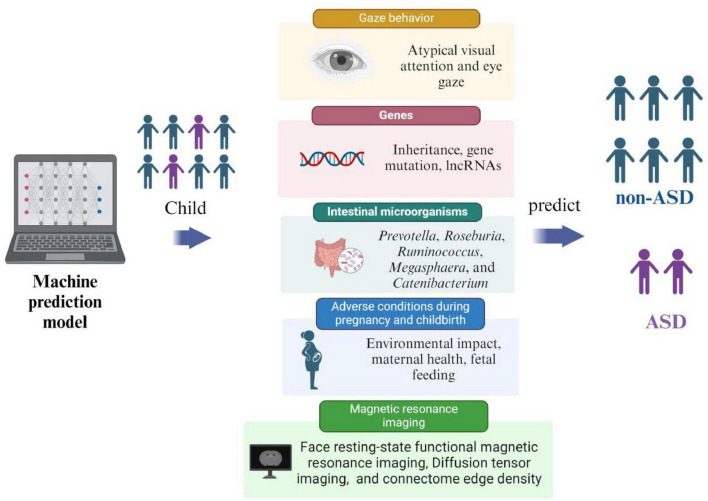
Factors in predictive models for ASD.

ASD is an irreversible mental development disorder. Its diagnosis relies on scales and behavioral tests, which can only identify patients with obvious symptoms. However, these tools often fail to accurately diagnose individuals with atypical or subtle presentations, limiting opportunities for early treatment and reducing intervention effectiveness. Early intervention can effectively prevent the onset of ASD, with a scientific and feasible risk prediction model serving as the foundation for predicting ASD. Rahman et al. (2020) used the electronic medical records (EMRs) system of the Israeli Health Maintenance Organization to extract data on ASD children and non-ASD children born from January 1, 1997 to December 31, 2008, and predicted ASD through an ML model. By incorporating parents’ sociodemographic information, medical history, and prescription medications data to train various ML algorithms, including Multiple Logistic Regression (MLR), Artificial Neural Network (ANN), and Random Forest (RF), they achieved a c statistic (the c statistic is mainly used to evaluate the prediction model’s accuracy; accuracy increases as the value gets closer to 1) of 0.709 for predicting ASD. The model demonstrated a sensitivity of 29.93%, specificity of 98.18%, accuracy of 95.62%, false positive rate of 1.81%, and positive predictive value of 43.35%. The study concluded that the ML algorithm, combined with EMR data, effectively identified ASD risk in early life and revealed previously unknown features associated with ASD risk. These methods can have the potential to enhance the accurate and effective detection of ASD in a large population of children. Currently, there is no universally accepted gold standard for ASD risk prediction models. Factors used for prediction include starting behavior (early behavioral indicators), magnetic resonance imaging, adverse factors affecting pregnancy, genetics, and gut microbiota. The development of ML-based risk prediction models for ASD is an ongoing area of research (Figure 3).

### 3.1 ASD ML prediction models based on genetic factors

The development of ASD is linked to genetic factors. At present, ML prediction models are increasingly used for genetic risk prediction (Yao et al., 2021). A study used a method based on ML to predict ASD risk genes, analyzing human brain spatiotemporal gene expression patterns, gene level constraint indicators, and other gene variation characteristics to predict the risk of ASD (Lin et al., 2020). Duda et al. (2018) constructed an ML model to rank ASD risk genes across the entire genome using a brain-specific functional relationship network (FRN) of genes. They identified some key pathways in early neural development of ASD through functional enrichment analysis of candidate gene networks. A new algorithm, Detecting Association With Networks (DAWN), was developed to identify ASD using rare variations in exome sequencing and gene co-expression in the fetal middle prefrontal and motor somatosensory neocortex. The algorithm converts the integrated data into a hidden Markov random field, where the structure of the graph is determined by gene co-expression, and combines these interrelationships with node-specific observations, namely, gene identity, expression, and genetic data, to estimate risk. DAWN was used to study the emerging ASD sequence data and gene expression data from other brain regions and tissues (Liu et al., 2014). ASD is a complex brain disorder with polygenic etiology. Wang and Avillach (2021) used a deep learning gene classifier to diagnose ASD, performing chi-square test on the collected genome data to extract common variants that may be protective or pathogenic to ASD and designing a diagnostic classifier based on Convolutional Neural Networks (CNNs) to predict ASD using the significant common variants identified. The deep learning model outperformed shallow ML models, achieving an area under the receiver operating characteristic curve of 0.955 and an 88% accuracy in identifying non-ASD individuals.

The prediction model develops constantly updated and developed. A new multi-label classification (MLC) model has been used to identify ASD risk genes and toxic chemicals on a large-scale dataset. First, the characteristic matrix and partial marker network of ASD risk genes and toxic chemicals were constructed from multiple heterogeneous biological databases. Based on global and local metrics, simulation results showed that the model has better classification performance than other MLC methods (Huang et al., 2021). This work will help promote the relationship discovery of ASD risk genes–environment interactions and aid in studying the gene–environment interactions in the future (genetic factors are internal, while environmental factors are external) to better understand the pathological mechanism of ASD. Wang and Wang (2020) developed a new ML method to predict candidate lncRNAs related to ASD. In the pre-training stage of model construction, an autoencoder network utilized gene expression data for representation learning, and random-forest-based feature selection was applied to the transcript-sequence-derived k-mers. The model includes LR, Support Vector Machine (SVM), and RF algorithms demonstrating the robustness of candidate priorities based on 10-fold cross-validation and hypothesis sites. These models are used to predict and prioritize a series of candidate lncRNAs (These lncRNAs demonstrate significant correlations with various ASD-related traits, such as sex differences, synapsin function, birth weight, and both intelligence and cognitive performance.), including some reported cis-regulators of known ASD risk genes. Gene mutations associated with ASD are polygenic, with a high frequency of gene mutations in humans. However, the relative contribution of each gene is small, hampering their identification. The details of the above ASD ML prediction models based on genetic factors are shown in Table 1.

**TABLE 1:** ASD ML prediction models based on genetic factors comparative table.

Model	Risk genes	Samples	Accuracy	Limitations	Ref
The gradient boosted trees	*NBEA*, *HERC1*, *TCF20*, *MYCBP2* and *CAND1*	The gene set comprised 121 positive genes and 963 negative genes.	AUC = 0.86	The ML model was constrained by a limited sample size and a small number of predictive variables.	(Lin et al., 2020)
The random forest	*BRAF*, *CACNA1C*, *CDKL5*, *CHD7*, *DMD*, *GATM*, *KCNJ10*, *NIPBL*, *OCRL*, *PTPN11*, *SGSH*, *AKAP9* and *TCF7L2*	Over 20,000 genes through Bayesian network integration of a diverse set of functional genomic data types derived from human, mouse and rat experiments.	AUC = 0.88	The ML model was constrained by a limited sample size and a small number of predictive variables.	(Duda et al., 2018)
Detecting Association With Networks(DAWN)	*CUL3*, *DYRK1A*, *GRIN2B*, *POGZ*, *SCN2A*, *TBR1*, *L1CAM*, *PTEN*, *STXBP1*, *MBD5*, *SHANK2*, *BBS10*, *FOXP1*, *TBL1XR1*, *ARID1B* and *ADNP*	Periods 3–5 and 4–6 with 10 and 14 brains were 107 and 140 replicates of expression per gene, respectively	–	They might not be captured effectively by current exome sequencing methods.	(Liu et al., 2014)
A convolutional neural network–based	*ARSD*, *MAGEB16*, and *MXRA5* genes	The ASD data set from the Simons Simplex Collection	AUC = 0.96	The ML model was constrained by a limited sample size and the prediction accuracy of its algorithm	(Wang and Avillach, 2021)
A multilabel convolutional neural network (MLCNN).	*FBXL7*, *SEMA5A*, *FAT1*, *CDH8*, *NXPH1*, *UPF3B*, *PLN*, *NR3C2*, *CTTNBP2*, *PTCHD1*, *FOXP1*, *GLRA2*, *BCL6*, *ANK1*, *PTHLH*, *RORA*, *CDH5*, *SLIT3*, *ARX*, *BTN2A1*, *SACS*, *HCCS*	The top-20 predicted risk genes have been demonstrated to have associations with ASD	AUC = 0.81	The ML model was constrained by a limited sample size	(Huang et al., 2021)
Logistic regression (LR), support vector machine (SVM) and random forest (RF) models	ENSG00000229807, ENSG00000240801, ENSG00000228971, ENSG00000258283, ENSG00000221857	The ASD-associated lncRNAs training dataset consisted of 366 ASD risk genes as positive instances and 1,762 non-ASD disease genes as negative instances.	AUC = 0.82	The ML model needs to obtain ASD clinical samples for predictive training to improve diagnostic accuracy.	(Wang and Wang, 2020)

### 3.2 ASD ML prediction model based on staring behavior

ASD is typically associated with atypical visual attention, and eye gaze data can be collected at a very young age. An automatic screening tool based on eye gaze data can identify the risk of ASD, which provides an opportunity to intervene before all symptoms manifest (Jiang et al., 2020, Tsuchiya et al., 2021). Using collected videos of Bangladeshi children from Dhaka Shishu Children’s Hospital and utilizing ML raters to determine the “risk score” of children’s ASD, this study improved the practicality and performance of the model. This was achieved by developing and applying a powerful new adaptive aggregation technology and establishing two classification layers. In the first layer, typical and atypical behavior are distinguished, while in the second layer, ASD and non-ASD are distinguished. Each layer utilizes a unique rater weighting scheme to summarize their classification scores based on the professional knowledge of different raters. Area under the receiver operating characteristic curve (AUC) was used to measure the accuracy of the model. The AUC of the first layer was 0.76, and that of the second layer was 0.85 (Tariq et al., 2019).

In addition, Liaqat et al. proposed two ML methods, the synthetic saccade method and the image-based method, to automatically classify children’s eye gaze data collected from natural image tasks. The first method uses the synthetic saccade pattern generation model to represent the baseline scan-path of a typical non-ASD individual. It combines this with the real scan-path and other auxiliary data as input for the depth learning classifier. The second method employs a more comprehensive image-based method, where the input image and a series of fixation maps are fed into a convolutional or recurrent neural network. In this experiment, the accuracy of ASD prediction in the validation dataset reached 62.13% (Liaqat et al., 2021). The details of the above ASD ML prediction models based on staring behavior are shown in Table 2.

**TABLE 2:** ASD ML prediction models based on staring behavior comparative table.

Model	Samples	Accuracy	Limitations	Ref
The best-fit diagnostic algorithm	222 individuals aged between 5 and 17 years were enrolled by physicians at seven research sites and affiliated clinics at Hamamatsu University School of Medicine, Hirosaki University, University of Fukui, Chiba University, Saga University, Kanazawa University, and Tottori University during a 6-month period beginning on 25 February 2018.	AUC = 0.84	The ML model was constrained by a limited sample size	(Tsuchiya et al., 2021)
The random forest	The Bangladesh Institute of Child Health, Dhaka Shishu Children’s Hospital recruit 150 children,including 50 with ASD, 50 with an speech and language conditions, and 50 with neurotypical development.	AUC = 0.76 and 0.85	Low accuracy of training data	(Grossi et al., 2016)

### 3.3 ASD ML prediction model based on risk factor exposure during pregnancy and childbirth

The incidence of ASD is linked to certain risk factors during pregnancy. Grossi et al. (2016) evaluated 27 potential risk factors related to postpartum and pregnancy. They collected data by interviewing the mothers of 45 ASD children and 68 typically developing children. After selecting 16 variables from the initial 27 variables through a TWIST dataset (input selection and test training) system, special artificial neural networks (ANNs) differentiated between ASD and control subjects, with an overall accuracy of 80.19%. The study suggests that exposure to adverse factors during gestation may induce gene mutations in offspring.

To identify newborns at risk of ASD and detect potential biomarkers shortly after birth, a retrospective study was conducted comparing biological measurements and ultrasound data from infants later diagnosed with ASD to those from Neurotypical (NT) infants. These data were originally collected during routine prenatal and postnatal care. A supervised ML algorithm incorporating cross-validation was employed to classify NT and ASD infants. When optimizing for a low false-positive rate, the model correctly identified 96% of NT infants and 41% of ASD infants, yielding a positive predictive value of 77%. The study identified several biomarkers associated with ASD, including sex, autoimmune diseases in maternal family, cytomegalovirus (CMV) infection, IgG CMV levels, timing of fetal head rotation, femur length during the third trimester, white blood cell count during the third trimester, fetal heart rate during labor, neonatal feeding patterns, and differences in body temperature at birth and the following day. Statistical analysis revealed that 38% of ASD-risk infants had significantly larger fetal head circumference compared to age-matched NT infants (Caly et al., 2021). Maternal stress and immune influence fetal brain development, highlighting the importance of the mother’s physical health during pregnancy for optimal fetal development. The details of the above ASD ML prediction models based on adverse conditions during pregnancy and childbirth are shown in Table 3.

**TABLE 3:** ASD ML prediction models based on adverse conditions during pregnancy and childbirth comparative table.

Model	Risk factors	Samples	Accuracy	Limitations	References
Artificial neural networks	solvents/paints occupational exposure during pregnancy, stressful events during pregnancy, pregnancy complications, perinatal complications, deficient breastfeeding after delivery, and early antibiotic therapy of the newborn.	The mothers of 45 ASD children and of 68 TD children. 24 siblings of 19 ASD children formed control group.	AUC = 0.80	The ML model was constrained by a limited sample size	(Grossi et al., 2016)
The gradient boosting decision tree algorithm	sex, maternal familial history of auto-immune diseases, maternal immunization to cytomegalovirus (CMV), IgG CMV level, timing of fetal rotation on head, femur length in the 3rd trimester, white blood cell count in the 3rd trimester, fetal heart rate during labor, newborn feeding and temperature diference between birth and 1 day after.	In 2012–2013, 5356 babies were born in the maternity Hospital of the University of Limoges in France. Two to five years later, 65 of these babies (1.21%) were diagnosed with ASD. The 63 babies with ASD (12 girls and 51 boys) were matched with 189 neurotypical (NT) babies based on mother’s age, parity and term of childbirth.	F_0.5_ = 0.63	The small sample size and the number of girls limit the generaliz ability of the results.	(Caly et al., 2021)

### 3.4 ASD ML prediction model based on brain MRI

Magnetic resonance imaging (MRI) is a non-invasive detection method with high spatial resolution and high density resolution, which can effectively reflect brain lesions. With the advancement of computer technology and medical imaging technology, the “risk prediction model” based on medical imaging big data has emerged. The occurrence of infantile autism was predicted using the segmentation and segmentation maps of sMRI, with a peak sensitivity of 73.1% and a peak specificity of 75.9% (Gao et al., 2021). Liu et al. (2020) attempted to construct a reproducible and robust ASD neural patterns using heterogeneous multi-site brain imaging datasets. Brain connectivity was assessed using face resting-state functional magnetic resonance imaging (fMRI) data from the CC200 atlas based on the Extra-Trees algorithm. Through a cross-validation strategy, the mean classification accuracy of this method was found to be 72.2% (sensitivity, 68.6%; specificity, 75.4%). It improves the accuracy of ASD prediction by about 2% and the specificity by 3.2%. The connectivity analysis of the optimal model highlights the brain regions that play a significant role in social cognition and interaction, revealing that the correlation between the anterior and posterior default mode networks (DMNs) of individuals with ASD is lower than that of the control group. This observation is consistent with previous studies, which enables this method to effectively identify individuals with ASD risk. In addition, utilizing a hierarchical structure, deep ML models can identify ASD based on interactions within hierarchical functional brain networks (FBNs) inferred from fMRI, achieving a classification accuracy of 82.1% (Qiang et al., 2023).

There is evidence that microstructural disorders and the disruptions in the connectome of white matter (WM) are related to the onset of ASD. The influence of age on the microstructure of WM was evaluated using Diffusion Tensor Imaging (DTI) and connectome Edge Density (ED) in ASD and control patients of different age groups. Fractional anisotropy (FA), Mean Diffusivity (MD), Radial Diffusivity (RD), Axial Diffusivity (AD), and ED maps were created for each subject. Voxel-wise and tract-based analysis was conducted using different combinations of improved ML classifiers and dimension reduction algorithms. The results showed that changes in the corpus callosum and connectome are related to ASD and are not present in infants and toddlers, but become more apparent in adolescents and young adults (Weber et al., 2022).

### 3.5 ASD ML prediction model based on intestinal microorganisms

There is a large number of bacteria, viruses, and fungi present in the human intestine, collectively known as gut microbiota. The normal human intestinal microorganisms are found in a state of dynamic equilibrium. When these microorganisms are disturbed, it can lead to disturbances in the normal functions of the digestive system, respiratory system, immune system, nervous system, and other bodily systems (Bose et al., 2020, Wastyk et al., 2021, Fu et al., 2021, Liu et al., 2022, Sun et al., 2024). Although the pathogenesis of ASD is not clear, individuals with ASD often experience gastrointestinal (GI) co-morbidities, such as irritable bowel syndrome, diarrhea, and chronic constipation, and the severity of these symptoms is linked to the degree of GI microbiota dysbiosis (Sanctuary et al., 2019, Harris et al., 2021). In addition, imbalances in intestinal microbiota is associated with the onset and progression of ASD (Dan et al., 2020, Fouquier et al., 2021, Li et al., 2022). Intestinal microbiota of ASD patients exhibit a reduction in alpha diversity (Lou et al., 2021). The abundance of *Bifidobacterium* spp. in the intestine of children with ASD is lower (Jendraszak et al., 2021). In a mouse model of ASD induced by SHANK gene deletion, changes in intestinal microbiota were observed, with an increase in *Firmicutes* and a decrease in *Proteobacteria* and *Verrucomicrobia*, as well as the presence of *Deferriactors*, *Tenericites*, and *Chlamydiae* (Sauer et al., 2019). Aseptic mice transplanted with intestinal microbiota from ASD patients exhibited increased repetitive behavior, decreased movement, and reduced communication (Sharon et al., 2019). Microbial transfer therapy has shown significant improvement in gastrointestinal and behavioral symptoms (Arnold et al., 2019, Kang et al., 2019, Kang et al., 2020, Li et al., 2021). In addition, supplementation with probiotics, such as *Lactobacillus* (L.) *reuteri* has been shown to improve social behavior in ASD mice through the vagus nerve (Sgritta et al., 2019).

Intestinal microbiota has shown significant predictive ability in disease prediction models. ML modeling of the human microbiome has the potential to identify microbial biomarkers and aid in the diagnosis of various diseases, including inflammatory bowel disease, diabetes, and colorectal cancer (Topçuoğlu et al., 2020). Midani et al. (2018) developed an ML model based on intestinal microbiota that, along with known clinical and epidemiological risk factors, can predict *vibrio cholerae* infection. Braun et al. (2019) analyzed a study on Crohn’s disease (CD) using a binary/ternary/ratio (BTR) model and found that the microbial richness of patients with CD in clinical, biomarker, and mucosal remission was significantly reduced, while the ecological imbalance index was significantly increased. Verhaar et al. (2022) used an ML model to study the relationship between intestinal microbiota composition and AD biomarkers in patients with AD, mild cognitive impairment (MCI), and subjective cognitive decline (SCD). Microbiota composition showed the best performance in predicting amyloid and p-tau levels using ML, with AUC values of 0.64 and 0.63, respectively. Intestinal microbiota is not only associated with many gastrointestinal diseases but also affects other parts of the body, as evidenced by its role in ASD. Although it has been demonstrated that the number and structure of intestinal microbiota may be closely related to ASD, there are few models based on intestinal microbiota to predict ASD. A recent study used a two-dimensional cellular automatic mechanism to build a mathematical model to simulate the growth rate and intestinal nutrients of *bifidobacteria, clostridium*, and *desulfovibrio*, as well as the growth rate and interaction after *lysozyme* was introduced into the intestine. The model simulation revealed that altering the number of *Clostridium* in the intestine could cause changes in the intestinal microbiota, potentially affecting the risk of ASD (Nagaraju et al., 2019). In a study by Wu et al. (2020), 297 subjects from the sequence read archive database were evaluated, including 169 ASD patients and 128 neurotypical subjects. Various analyses were conducted, including α-diversity, phylogenetic profiles, and functional profiles. Principal component analysis (PCA) showed that ASD and neurotypical subjects could be distinguished based on unweighted UniFrac distance. Through linear discriminant analysis effect size (LEfSe) evaluation and random forest analysis, *Prevotella*, *Roseburia*, *Ruminococcus*, *Megasphaera*, and *Catenibacterium* were identified as potential biomarkers of ASD. Functional analysis revealed six significant pathways distinguishing ASD and neurotypical subjects, including oxidative phosphorylation, nucleotide excision repair, peptidoglycan biosynthesis, photosynthesis, photosynthesis proteins, and two-component system. Based on these changes in the intestinal microbiota of ASD subjects, four ML models were developed: RF, MLP, kernelized Support Vector Machines (SVMs) with the RBF kernel, and Decision Tree (DT). Among them, the RF model demonstrated the best performance achieving an F1 score (a measure of a model’s precision and recall) of 0.74 and an AUC of 0.827, indicating both reliability and generalizability. The details of the above ASD ML prediction models based on intestinal microorganisms are shown in Table 4.

**TABLE 4:** ASD ML prediction models based on intestinal microorganisms comparative table.

Model	Risk factors	Samples	Accuracy	Limitations	Ref
The SVM to a recursive feature elimina tion (RFE) algorithm	*Vibrio cholerae*	A stool culture positive for *vibrio cholerae* O1 as the sole pathogen. other contacts within 6 hours of presentation of the index case as control at the International Center for Diarrhoeal Disease Research, Bangladesh	AUC = 0.80	The sample size is small and not representative	(Midani et al., 2018)
The random forest	*Christensenellaceae* and *S24.7*	in a prospective observational cohort of patients with quiescent Crohn’s disease (45 cases, 217 samples) over 2 years or until clinical flare occurred	AUC = 0.78	The sample is not representative	(Braun et al., 2019)
The logistic regression	*Leptum*, *ventriosum group* spp., *Lachnospiraceae* spp., *Marvinbryantia* spp., *Monoglobus* spp., *torques group* spp., *Roseburia hominis*, *Christensenellaceae R-7* spp., *Lachnospiraceae* spp., *Lachnoclostridium* spp., *Roseburia hominis* and *Bilophila wadsworthia*	170 patients from the Amsterdam Dementia Cohort, including 33 with Alzheimer’s disease dementia, mini-mental state examination (MMSE), 21 with mild cognitive impairment and 116 with subjective cognitive decline	AUC = 0.64 and 0.63	The sample is not representative	(Verhaar et al., 2022)
2-D Cellular Automaton	*Bifidobacterium*, *Clostridium* and *Desulfovibrio*	–	–	There are few types of bacteria detected by the model	(Nagaraju et al., 2019)
The random forest	*Prevotella*, *Roseburia*, *Ruminococcus*, *Megasphaera*, and *Catenibacterium*	297 subjects from the Sequence Read Archive database, including 169 individuals with ASD and 128 neurotypical subjects	AUC = 0.827	The sample size is small and not representative, and the model still needs to be optimized	(Wu et al., 2020)

## 4 Conclusion and perspectives

The research outlined above has demonstrated the effectiveness of the ML model in predicting ASD using genetic background, gaze behavior, adverse perinatal conditions, brain MRI data, and intestinal microbiota. These approaches play a crucial role in forecasting the onset and progression of ASD. However, several improvements are required for enhancing the ML prediction model: (1) expanding the sample size and predicting factors for ML; (2) unifying the selection of prediction factors and detection methods; (3) continuously optimizing, training, and testing ML models to improve prediction accuracy; and (4) conducting a crowd cohort study in order to verify the accuracy and feasibility of the machine model.

The integration and development of ML and prediction models have been increasingly applied in the medical field (Lee et al., 2022, Ben-Yacov et al., 2023). ML prediction models for ASD have also been developed both domestically and internationally (Qiu et al., 2020, Zhu et al., 2022). This approach presents great challenges and potential for more effective screening, diagnosis, and treatment in clinical practice.

ML models discussed in the literature primarily serve two key purposes:

(1) Feature selection, involving the extraction of the most discriminative features from the data to be used in prediction or as a foundation for training ML models;
(2) Classification of ASD and control groups, enabling the identification of differential features between both populations and facilitating the detection of ASD-related characteristics in previously unknown samples.

Most of the presented studies employed traditional ML techniques, such as SVM, LR, RF, and DT. Among these techniques, RF was the most frequently utilized due to its superior performance compared to the other techniques and its relatively low computational complexity during training. However, neural network models were solely used, with only classic architectures such as convolutional neural networks, multilabel convolutional neural networks, and artificial neural networks, being applied. It is evident that the discussed intestinal microbiota prediction models mainly rely on conventional ML algorithms, including RF, SVM, recursive feature elimination algorithm, and LR. However, AUC values for these models range from 0.64 to 0.83, indicating that they have not yet reached optimal performance. To improve model performance, experience-based algorithms incorporating meta-heuristic techniques can be employed for intelligent parameter optimization during model tuning. This approach not only improves predictive accuracy but also effectively mitigates the risk of overfitting, especially when considering small sample sizes. Future research will focus on exploring interpretable AI models and validating the proposed methods on additional ASD datasets as they become available, before being applied in clinical settings.

Currently, there is limited research on artificial intelligence in predicting ASD risk through intestinal microbiota analysis, and a comprehensive system has not been established yet. The main problem with ML is the small sample size and the lack of inclusion of key high-risk factors, which limits the diversity and accuracy of predictive factors. It is hoped that further research on ML models, especially the combination of laboratory research and population cohort studies, can achieve the following goals:

(1) Establishing a library of ASD intestinal microbiota samples, identifying the target bacteria in the microbiota as biomarkers using machine models, determining disease risk through screening these bacteria, and establishing a prediction model for early disease detection.
(2) Screening high-risk bacteria through ML prediction models, formulating standardized, refined, and intelligent treatment measures tailored to the characteristics of ASD patients, establishing more precise diagnostic standards and medication guidelines, and providing insights for precision medical treatment.
(3) Utilizing artificial intelligence to predict intestinal microbiota patterns as a guide for the prediction and treatment of ASD, providing a reference for the development of targeted drugs to regulate microbiota, and reducing the time and cost of drug development.
(4) Recognizing the deficiencies in underdeveloped countries medical services within the broader context of global healthcare disparities, particularly the low professional standards of doctors, which reflect systemic challenges in resource allocation, education, and infrastructure development in underserved regions worldwide. This issue underscores the need for a more equitable and inclusive approach to healthcare that addresses both local and global inequities, ensuring that all individuals, regardless of their geographic location, have access to quality medical care and professional expertise.

Early prediction, diagnosis, and treatment of ASD are crucial for enhancing neurodevelopmental outcomes in children. Gut microbiota plays a crucial role in the pathogenesis of ASD through various mechanisms, including the regulation of metabolites, modulation of immune responses, and activation of neural signaling pathways. The dynamic variations in gut microbiota present potential targets for early prediction and intervention in ASD. However, the translation of basic research into clinical practice encounters several challenges, such as methodological limitations, data integrity issues, and ethical considerations. Addressing privacy, data deficiency and ethical issues in population surveys is not a matter of a single technical approach, but a systematic project that runs through the entire research life cycle and integrates legal compliance, technical safeguards, ethical principles and scientific rigor. Always placing the rights, dignity and well-being of participants at the core is the cornerstone of ensuring that research is scientifically effective and responsible. Moreover, the integration of multidisciplinary technologies, including single-cell omics and AI analytics, is essential for explaining the complex networks involved in gut-brain interactions.

Future research should focus on the development of microbiota-based diagnostic tools and precision therapeutic strategies that selectively target specific microbial communities. Such approaches hold significant promise for improving neurodevelopmental outcomes for individuals with ASD.
